# A new genus of temperate woody bamboos (Poaceae, Bambusoideae, Arundinarieae) from a limestone montane area of China

**DOI:** 10.3897/phytokeys.109.27566

**Published:** 2018-10-12

**Authors:** Yu-Xiao Zhang, Peng-Fei Ma, De-Zhu Li

**Affiliations:** 1 Yunnan Academy of Biodiversity, Southwest Forestry University, Kunming, Yunnan 650224, China Kunming Institute of Botany, Chinese Academy of Sciences Kunming China; 2 Germplasm Bank of Wild Species, Kunming Institute of Botany, Chinese Academy of Sciences, Kunming, Yunnan 650201, China Southwest Forestry University Kunming China

**Keywords:** *
Ampelocalamus
*, climbing bamboos, *
Hsuehochloa
*, new genus

## Abstract

*Ampelocalamuscalcareus* is a climbing and slender bamboo, known from south Guizhou, China. This species grows in broadleaved forests of limestone montane areas. Recent molecular phylogenetic analyses demonstrated that *A.calcareus* was sister to all other lineages of the tribe Arundinarieae rather than a member of *Ampelocalamus*. The morphological features and habitats of *A.calcareus* and related genera including *Ampelocalamus*, *Drepanostachyum* and *Himalayacalamus* were compared and discussed. The characteristics of the branch complements, nodes and foliage leaves distinguish *A.calcareus* from morphologically similar taxa. On the basis of molecular and morphological evidence, we propose to establish a new genus, *Hsuehochloa*, to accommodate *A.calcareus* and to honour the late Chinese bamboo taxonomist Chi-Ju Hsueh (Ji-Ru Xue). In addition, we describe the inflorescence of *Hsuehochloa* for the first time.

## Introduction

Temperate woody bamboos or the tribe Arundinarieae (Bambusoideae, Poaceae) comprise approximately 550 species in 31 genera (BPG 2012, Clark et al. 2015). They are mainly distributed in temperate to subtropical montane areas of East Asia (Ohrnberger 1999) with China as the centre of species diversity (Li et al. 2006), but also in Southeast Asia, south India, Sri Lanka, North America and Africa.

The recent plastid molecular phylogenetic results indicated that there were 12 major lineages in temperate woody bamboos, i.e. I. *Bergbambos*, II. *Oldeania*, III. *Chimonocalamus*, IV. *Shibataea* clade, V. *Phyllostachys* clade, VI. *Arundinaria* clade, VII. *Thamnocalamus*, VIII. *Indocalamuswilsonii*, IX. *Gaoligongshania*, X. *Indocalamussinicus*, XI. *Ampelocalamuscalcareus* and XII. *Kuruna*. However, relationships amongst them remain largely uncertain (Triplett and Clark 2010, Zeng et al. 2010, Yang et al. 2013, Attigala et al. 2014, 2016, Ma et al. 2014, Zhang et al. 2016, Zhang et al. 2017). Those lineages are strongly inconsistent with the morphological classification at the generic and subtribal levels (Keng and Wang 1996, Li 1997, 1999, Ohrnberger 1999). Most species and genera were nested within lineages IV, V and VI, while some lineages included only one species (lineages I, VIII, IX, X, XI). Lineages I and IX consisted of *Bergbambostessellata* (Nees) Stapleton and *Gaoligongshaniamegalothyrsa* (Handel-Mazzetti) D. Z. Li, Hsueh & N. H. Xia, respectively and *Bergbambos* Stapleton and *Gaoligongshania* D. Z. Li, Hsueh & N. H. Xia are both monotypic (Li et al. 1995, Stapleton 2013). Lineages VIII and X were formed by *Indocalamuswilsonii* (Rendle) C. S. Chao & C. D. Chu and *I.sinicus* (Hance) Nakai, respectively, with *I.sinicus* as the lectotype of the genus *Indocalamus* Nakai. *Ampelocalamuscalcareus* C. D. Chu & C. S. Chao (lineage XI) was recovered as the sister taxon to all the other temperate woody bamboos (Yang et al. 2013, Ma et al. 2014). The phylogenetic positions of the abovementioned five monotypic lineages have also obtained some support from nuclear gene trees (Zhang et al. 2012, Yang et al. 2013).

Molecular phylogenetic results provide fresh perspectives for taxonomy, especially for lineages VIII, X and XI with only one species. Continuing to include these bamboos in the present genera renders these genera polyphyletic and causes problems when describing or citing them. In this paper, we propose to establish a new genus for *Ampelocalamuscalcareus* based on morphological characters and previous molecular results. For the other two monotypic lineages (VIII and X), taxonomic revisions will be made in a separate paper.

## Materials and methods

*Drepanostachyum* P. C. Keng and *Himalayacalamus* P. C. Keng are morphologically close to *Ampelocalamus* S. L. Chen, T. H. Wen & G. Y. Sheng (Li et al. 1996). These three genera all have pachymorph rhizomes, prominent or conspicuous nodal sheath scars and pendulous culms. Sometimes it is difficult to see the difference when only the vegetative features are available. Some species of *Ampelocalamus* were transferred from the genus *Drepanostachyum* (Keng and Wang 1996, Stapleton et al. 2005, Li et al. 2006) and several taxa of *Drepanostachyum* were combined into *Himalayacalamus* (Stapleton 1994). It is necessary to compare characters of *Ampelocalamuscalcareus* with those two genera in order to clarify their morphological similarities and differences.

### Specimen examination

The type specimen of *Ampelocalamuscalcareus* was examined at the herbarium of Nanjing Forestry University (NF). We also examined specimens of *A.calcareus*, other species of *Ampelocalamus*, *Drepanostachyum* and *Himalayacalamus* at herbaria of Kunming Institute of Botany, Chinese Academy of Sciences (KUN), Nanjing University (N), Institute of Botany, Chinese Academy of Sciences (PE) and Sichuan Agricultural University, Dujiangyan Campus (SIFS) (specimens of N and PE were checked through the website http://www.cvh.ac.cn/).

### Living plant observation

In 2010, one clump of *Ampelocalamuscalcareus* was introduced by P. F. Ma and Z. M. Cai from Libo, Guizhou and cultivated at the greenhouse of the Germplasm Bank of Wild Species (GBOWS), Kunming Institute of Botany, Chinese Academy of Sciences, Kunming. This introduced clump flowered between 2012 and 2013. Unfortunately, the clump did not grow well and only a small piece of inflorescence was collected before it died. After fieldwork in early 2015, it was confirmed that the wild populations also flowered and died during the same period. More seedlings were introduced into the greenhouse of GBOWS at Kunming by C. Guo and Y. Guo in March 2015. They grew well in the greenhouse and became mature individuals after three years. Vegetative features including culms, culm sheaths, branch complements and foliage leaves were observed based on those individuals.

### Morphological comparison

Four reproductive characters and 15 vegetative characters were selected and compared across *Ampelocalamuscalcareus*, *Ampelocalamus*, *Drepanostachyum* and *Himalayacalamus*. For *A.calcareus*, the morphological data were observed and obtained based on herbarium specimens, living plants and literature. We observed and measured the structure of the inflorescence of *A.calcareus* by hand lens (30×) and stereomicroscope (Leica M166FC) without dissecting the spikelet due to the scarcity of materials. For the other genera, the morphological data were obtained from specimens and literature. The habitats of *Ampelocalamuscalcareus*, *Ampelocalamus*, *Drepanostachyum* and *Himalayacalamus* were also compared based on literature.

## Results

Morphological characteristics and habitat data are summarised in Table 1. *Ampelocalamuscalcareus* and species of *Ampelocalamus*, *Drepanostachyum* and *Himalayacalamus* are all unicaespitose. Culms of *A.calcareus* are procumbent or scrambling, while culms of *Ampelocalamus*, *Drepanostachyum* and *Himalayacalamus* are pendulous or seldom scrambling. The characteristics of culm sheaths, internodes, branch complements, nodal sheath scars and foliage leaves are variable across *A.calcareus*, *Ampelocalamus*, *Drepanostachyum* and *Himalayacalamus*.

The inflorescence of *Ampelocalamuscalcareus* is semelauctant and racemose. The spikelet has five florets and the floret possesses a purple-green lemma (ca. 1 cm long), palea shorter than the lemma (ca. 0.8 cm long), three purple stamens (4 mm long) and two plumose stigmas. The inflorescence of *Ampelocalamus*, *Drepanostachyum* and *Himalayacalamus* has been described in detail in other literature (e.g. Stapleton 1994, Li et al. 2006), therefore, we only list some key features in Table 1.

Analysis of the habitat data demonstrates that *Ampelocalamuscalcareus* mainly occurs under broadleaved forests of limestone areas below 1000 m; other *Ampelocalamus* species grow under broadleaved forests, on stony slopes (limestone, granite or basalt) and riverside slopes usually from 200 m to 1800 m alt.; taxa of *Drepanostachyum* are usually distributed under coniferous and broadleaved mixed forests from 1300 m to 3200 m alt.; species of *Himalayacalamus* occur under temperate forests from 1200 m to 3000 m alt. (Table 1).

## Discussion

**Figure 1.**
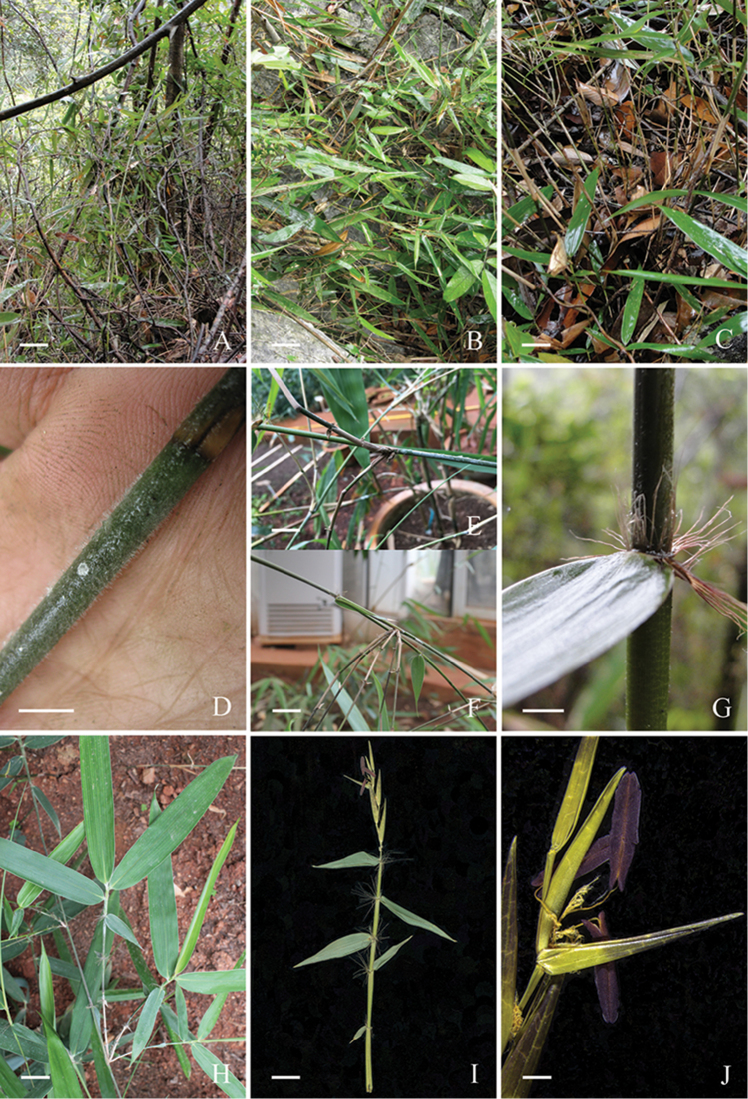
*Hsuehochloacalcarea*. **A, B** Habit and habitat **C** Clump **D** Young culm with white pubescence **E, F** Branch complement **G** Culm sheath **H** Leaves **I** Inflorescence **J** Floret (**A–D, G** from *P. F. Ma & Z. M. Cai 10050***E, F, H** from seedlings introduced from Libo, Guizhou, China **I, J** from *P. F. Ma s.n*). Scale bars: 5 cm (**A–C**); 0.5 cm (**D, G**); 2 cm (**E, F**); 1 cm (**H, I**); 1mm (**J**).

*Ampelocalamuscalcareus* was described by Chao and Chu (1983) based on vegetative specimens. This species has pachymorph rhizomes with short necks and apically drooping culms (Fig. 1) that are similar to other species of the genus *Ampelocalamus*, especially to the type species *A.actinotrichus* (Merrill & Chun) S. L. Chen, T. H. Wen & G. Y. Sheng. Moreover, the conspicuous auricles and radiate oral setae on the culm sheath and leaf sheath are similar to *A.actinotrichus* as well. However, characteristics of nodes, branch complements and leaf blades are quite different from *Ampelocalamus*. *Ampelocalamuscalcareus* has inconspicuous nodal sheath scars, a solitary branch at the base and 3–7 subequal branches at the middle and upper parts of the culm and leathery leaf blades. Other taxa in *Ampelocalamus* usually possess prominent nodal sheath scars with a corky collar, many branches with a central dominant one that may replace the culm and papery leaf blades. Branches at the nodes of *A.calcareus* are long (50–100 cm), pendulous and nearly as thick as the culm, which makes culms scrambling or procumbent. There are also some other vegetative features that can distinguish *A.calcareus* from typical *Ampelocalamus* species, as summarised in Table 1.

Culms of *Drepanostachyum* and *Himalayacalamus* are distally pendulous, but not scrambling, which is different from *Ampelocalamuscalcareus*. Branches on mid-culms of *Drepanostachyum* and *Himalayacalamus* are usually more than 15 in number and subequal without a central dominant one, while *A.calcareus* has no more than 10 subequal branches. Culm sheaths of *Drepanostachyum* and *Himalayacalamus* are usually deciduous and glabrous abaxially, whereas culm sheaths of *A.calcareus* are persistent and densely white pubescent abaxially. *Ampelocalamuscalcareus* has conspicuous auricles and oral setae on culm sheaths and leaf sheaths and ovate-lanceolate culm blades, while auricles and oral setae are often absent and culm blades are subulate or linear in *Drepanostachyum* and *Himalayacalamus*.

Due to the incomplete nature of the flowering material (Fig. 1), the description and comparison provided in Table 1 may not be fully accurate for healthy individuals flowering in the wild. The type of inflorescence of *A.calcareus* is similar to *Himalayacalamus* (racemose); the number of florets per spikelet is similar to *Ampelocalamus* and *Drepanostachyum* (5 vs. 2–7); they all have three stamens and two plumose stigmas, but the anther colour of *A.calcareus* is purple while anthers are yellow in *Ampelocalamus*, *Drepanostachyum* and *Himalayacalamus*.

Through comparison of morphological characters, we conclude that *Ampelocalamuscalcareus* morphologically resembles species of *Ampelocalamus*, *Drepanostachyum* and *Himalayacalamus* in its pachymorph rhizomes and is especially similar to *Ampelocalamus* in its climbing habit. However, the branch complements and the characteristics of its nodes, culm sheaths and foliage leaves can distinguish this species from all taxa in these three genera. The inflorescence of *A.calcareus* is also similar to these three genera (on the basis of our incomplete material) in its semelauctant structure, the presence of three stamens and two stigmas.

Molecular phylogenetic studies indicated that *Ampelocalamus*, *Drepanostachyum* and *Himalayacalamus* had a close relationship in nuclear gene based phylogenies, although only limited taxa of those genera were sampled (Yang et al. 2013). Nonetheless, *Ampelocalamuscalcareus* was sister to all the other taxa of the tribe Arundinarieae in plastid and nuclear gene trees (Yang et al. 2013, Ma et al. 2014, Attigala et al. 2016, Zhang et al. 2016). The morphological similarity between the distantly related *A.calcareus* and those three genera (*Ampelocalamus*, *Drepanostachyum* and *Himalayacalamus*) demonstrated that morphological characters had undergone complex evolutionary trajectories in those taxa and also in the whole tribe and some important features in bamboo taxonomy were homoplastic or convergent that was illustrated in other studies of Arundinarieae and some tropical woody bamboos (Yang et al. 2008, Tyrrell et al. 2012, Attigala et al. 2016).

The habitat and altitude of *A.calcareus* are more similar to other typical species of *Ampelocalamus* than they are to *Drepanostachyum* and *Himalayacalamus* (Table 1).

Based on the above analysis of morphology, molecular phylogenetic relationships and habitat, we propose to establish a new genus to accommodate *Ampelocalamuscalcareus*.

**Table 1.** Comparison of morphological characters and habitats of *Ampelocalamuscalcareus* (= *Hsuehochloa*), *Ampelocalamus*, *Drepanostachyum* and *Himalayacalamus*.

	* Hsuehochloa *	* Ampelocalamus *	* Drepanostachyum *	* Himalayacalamus *
Clump form	Unicaespitose, drooping, procumbent or scrambling	Unicaespitose, pendulous or scrambling	Unicaespitose, pendulous	Unicaespitose, nodding to pendulous
Culm height (length)	4–6 m	3–10 m (usually 3–5 m)	1.5–5.4 m (usually 2–3 m)	2–9 m
Culm diameter	4–5 mm	5–15 (40) mm	7–25 mm	10–35 mm
Internode	Terete, densely white pubescent and white powdery, later subglabrous	Terete, finely ridged, usually glabrous	Terete, glabrous	Terete, glabrous
Branch complement	Solitary at the base, 3–7 at the middle and upper, subequal	Many (less than 20), geniculate, central often dominant	Numerous (15 to 80), verticillate, subequal, slender	Many (15 to 30), subequal, slender
Nodal sheath scar	inconspicuous	prominent, often with corky collar	prominent	prominent
Culm sheath	1/2 as long as the internode, persistent, densely white pubescent abaxially, glabrescent	Shorter than the internode, deciduous, often sparsely setose abaxially	Longer or shorter than the internode, deciduous or persistent, glabrous or sparsely setose abaxially, adaxially scabrous apically	Longer or shorter than the internode, deciduous, glabrous or seldom setose abaxially
Culm sheath auricle	Falcate, amplexicaul	Absent or minute (*A.actinotrichus* with prominent auricles)	Absent	Absent
Culm sheath oral setae	Several, radiate	Absent except *A.actinotrichus*	Absent	Absent
Culm sheath blade	Ovate-lanceolate, reflexed	Linear, linear-lanceolate, lanceolate, ovate-lanceolate, erect or reflexed	Subulate or linear, erect or reflexed	Subulate or linear, erect or reflexed, readily deciduous
Leaf number of the ultimate branch	2–5	3–11	3–5	3–7
Leaf sheath	Glabrous	Glabrous or pubescent	Glabrous	Glabrous
Leaf auricle	Falcate	Absent or present	Absent or minute	Absent or minute
Leaf oral setae	Several, radiate	Radiate when present	Absent or present	Absent or present
Leaf blade	Leathery, glabrous	Papery, glabrous or pubescent	Papery, glabrous	Papery, glabrous or abaxial midrib hairy proximally
Inflorescence	Racemes	Panicles	Panicles	Racemes
No. of florets per spikelet	5	2–7	2–6	1 or 2
Stamen	3, anthers purple	3, anthers yellow	3, anthers yellow	3, anthers yellow
Stigma	2, plumose	2, plumose	2, plumose	2, plumose
Habitat	Limestone montane areas, alt. 500–950 m	Broad-leaved forests, stony slopes (limestone, granite or basalt), riverside slopes, alt. 200–1800 m	Slopes, coniferous and broadleaf mixed forests, 1300–3200 m	Temperate forests, 1200–3000 m

## Taxonomic treatment

### Hsuehochloa

Taxon classificationPlantaePoalesPoaceae

D. Z. Li & Y. X. Zhang gen. nov.

urn:lsid:ipni.org:names:77190833-1

#### Diagnosis.

*Hsuehochloa* resembles genera *Ampelocalamus*, *Drepanostachyum* and *Himalayacalamus*, but differs from those genera by its thin culms (4–5 mm), fewer branches in each branch complement (1, 3–7), inconspicuous nodal sheath scar, falcate auricles and leathery foliage leaves.

#### Type.

*Hsuehochloacalcarea* (C. D. Chu & C. S. Chao) D. Z. Li & Y. X. Zhang, comb. nov. (77190834-1)

Basionym. *Ampelocalamuscalcareus* C. D. Chu & C. S. Chao, Acta Phytotax. Sin. 21: 204–206. 1983. Type: CHINA, Guizhou, Libo, 500 m, *C. D. Chu, C. S. Chao, J. Q. Zhang & K. M. Lan 81018* (holotype, NF!; isotype, PE!)

#### Description.

Rhizomes pachymorph. Culms caespitose, apically drooping, procumbent or scrambling, 4–6 m long, 4–5 mm in diameter, internodes terete, 8–18 cm long, densely white pubescent initially at the upper part, later subglabrous; nodes and sheath scars inconspicuous. Branch complements with one branch proximally and 3–7 branches apically, branches 0.5–1 m long, slender, subequal. Culm sheaths persistent, 1/2 as long as internodes, densely white pubescent, glabrescent, margins densely white ciliate; auricles falcate, amplexicaul; oral setae many, radiate, ca. 1 cm; ligule short, apex densely white fimbriate; blade reflexed, green, ovate-lanceolate. Foliage leaves 2–5 per ultimate branch; sheaths glabrous, glossy, margins ciliate; auricles present; oral setae deciduous, radiate, 5–7 mm; ligule short, apex long, white ciliate; blade 7–20 × 1.2–3 cm, thinly leathery, abaxially slightly glaucous, glabrous on both surfaces, secondary veins indistinct, 4–7 pairs. Inflorescence imperfectly known, semelauctant, racemose possibly with 1 or few spikelets; glumes not seen; florets 5; lemma ca. 1 cm long, purple green; palea ca. 0.8 cm long; lodicules not seen; stamens 3, anthers purple, 4 mm long; ovary and style not seen; stigmas 2, plumose.

#### Etymology.

*Hsuehochloa* was named in honour of the late Prof. Chi-Ju Hsueh (Ji-Ru Xue in *Pinyin* transliteration) (1921–1999), a pioneer Chinese botanist on bamboos of SW China and mentor of the senior author in 1983–1986. *Hsueh* stands for his family name and *chloa* means grass.

#### Distribution and habitat.

Endemic to south Guizhou, China, under broadleaved forests in a limestone montane area at 500–950 m altitude.

#### Additional specimens examined.

**CHINA.** Guizhou: Libo, 950 m alt., May 1982, *X. H. Song 919* (NF), *J. P. Ruan 90041* (N), 600–700 m alt., November 6 2006, *T. P. Yi 06093 & 06094* (SIFS), 679 m alt., 25°26.691'N, 107°56.823'E, 14 April 2010, *P. F. Ma & Z. M. Cai 10050* (KUN), 653 m alt., 25°25.783'N, 107°56.533'E, 28 March 2015, *C. Guo* & *Y. Guo GC 82* (KUN), 667 m alt., 25°25.7'N, 107°56.25'E, 16 May 2015, *X. Y. Ye & M. Y. Zhou YXY190* (KUN).Yunnan (Kunming): cultivated in the greenhouse of GBOWS, Kunming, 1900 m alt., January 2013, *P. F. Ma s.n.* (KUN).

## Supplementary Material

XML Treatment for Hsuehochloa
